# Hospital Enterprise

**Published:** 1897-09

**Authors:** 


### Hospital Enterprise.

A hospital in Omaha is distributing broadcast a catchy circular, which reads in part as follows: "Accident insurance is a good thing. Insurance against ordinary sickness is a better thing. Both in one is the best thing. Do not wait until sickness or accidents come to you before taking out a membership certificate entitling you to free admission, bed, board, nursing, medical and surgical care in case of accident or sickness in the Methodist Hospital or the Omaha Hospital and Deaconess Home Association of the Methodist Episcopal Church. A two-edged sword cuts both ways, and accomplishes its mission in either direction. So does your membership fee. If you have occasion to use the hospital as a member, you have the first right to accommodations, above any other class of patients, and you will be most tenderly cared for. If the Lord spares you in perfect health, your money will assist the institution in caring for some one else not able to pay. It thus becomes a sweet charity whose fragrant memory will follow you all your life." The cost of membrship is $10 a year in advance. The payment of $250 in advance makes one a life member entitled to continuous free treatment for himself or, "if the Lord spares him in perfect health," for some one else of whom Providence is not so careful.—*N. Y. Medical Record.*

